# The Psychological Pathway to Suicide Attempts: A Strategy of Control Without Awareness

**DOI:** 10.3389/fpsyg.2021.588683

**Published:** 2021-03-18

**Authors:** Vanessa G. Macintyre, Warren Mansell, Daniel Pratt, Sara J. Tai

**Affiliations:** ^1^Division of Psychology and Mental Health, School of Health Sciences, The University of Manchester, Manchester, United Kingdom; ^2^Manchester Academic Health Science Centre, Manchester, United Kingdom; ^3^Greater Manchester Mental Health NHS Foundation Trust, Manchester, United Kingdom

**Keywords:** suicide, suicidal ideation, suicide attempts, prevention, control, awareness

## Abstract

**Objectives:**

This paper aims to identify potential areas for refinement in existing theoretical models of suicide, and introduce a new integrative theoretical framework for understanding suicide, that could inform such refinements.

**Methods:**

Literature on existing theoretical models of suicide and how they contribute to understanding psychological processes involved in suicide was evaluated in a narrative review. This involved identifying psychological processes associated with suicide. Current understanding of these processes is discussed, and suggestions for integration of the existing literature are offered.

**Results:**

Existing approaches to understanding suicide have advanced the current knowledge of suicide in various ways. They have guided valuable research in the following areas: motivations for suicide and the psychological distress which influences suicide attempts; ambivalence about suicide; suicidal individuals’ focus of attention; and ways in which individuals who contemplate suicide differ from individuals who attempt suicide. We outline a new theoretical framework as a means to integrating all of these concepts into the three principles of control, conflict, and awareness. Within this framework, suicide is regarded as occurring due to a long standing conflict between an individual’s personal goals, culminating in an episode of acute loss of control. The new framework posits that the individual then strives to regain control through the means of suicide because of a narrowed awareness of consequences of their actions on other valued goals. This psychological mechanism of limited awareness is posited to be the common pathway by which individuals make a suicide attempt, regardless of which risk factors are present.

**Conclusion:**

This article introduces a theoretical framework that generates several hypotheses for future research, and focuses on psychological processes occurring during immediate crisis. One of the key hypotheses resulting from our predictions on how individuals progress from contemplating to attempting suicide will be tested in an ongoing program of research: Individuals who attempt suicide have a significantly reduced awareness of consequences of suicide, which would negatively impact on their important life goals, values, principles, or ideals, compared to individuals who contemplate suicide. Therapy guided by the new framework may be more flexible, immediate, and client-focused than other therapies for suicidal individuals.

## Introduction

Suicide is a leading cause of death worldwide (World Health Organization, 2016), and more research is needed on arguably the most important factor for intervention - the mechanism which causes individuals to make a suicide attempt (Klonsky and May, 2014). Theoretical models of suicide have made significant progress toward understanding suicide, including the circumstances when individuals attempt suicide (Klonsky et al., 2018). There is currently no common pathway to understanding the multiple types of interventions for suicide and their various mediating mechanisms. Current psychological interventions for suicidal individuals, which are informed by existing theoretical models, have limitations. Since they aim to address multiple risk factors for suicide such as entrapment and perceived burdensomeness, as recommended by existing models (Joiner, 2005; Rudd, 2006; Klonsky and May, 2015; O’Connor and Kirtley, 2018), this often entails a structured approach involving multiple sessions (Linehan et al., 1991; Jobes, 2006; Stanley et al., 2009; Tarrier et al., 2013). Subsequently, clients may have fewer opportunities to speak freely about their problems, and the adaptation of these interventions to settings where time and resources are limited, such as inpatient ward and prisons, may pose challenges. Lastly, existing theoretical models include risk factors which are not directly modifiable in treatment, such as family history of suicide and pain sensitivity (Klonsky and May, 2015; O’Connor and Kirtley, 2018).

This article will review the contributions of various theoretical models to the current understanding of suicide. It will also introduce a new theoretical framework to understanding suicide from ideation-to-action, as recommended in previous literature (May and Klonsky, 2016), and describe how the new framework can integrate the contributions of recent theoretical models. Lastly, we discuss ways in which this theoretical framework may be helpful in informing future research on understanding suicide; in particular, the mechanism underlying suicide attempts and in informing psychological interventions. Our theoretical framework is intended to complement and extend existing models of suicide, and therefore some of its constructs map onto theoretical concepts which are explained in existing theoretical models of suicide using other terms. However, the new integrative theoretical framework has a novel focus on a single pathway to suicide which is mediated by striving for control and goal conflict awareness, both of which will be explained in detail in Sections “A Framework for Understanding Suicide Informed by Perceptual Control Theory”, “Predisposing to a Crisis”, “Precipitating a Crisis”, and “Mediating Suicide Behaviors”. Foremost, this theoretical framework focuses on an important niche of when the client is in immediate crisis, and intervention around this time. A simple and effective intervention for crisis periods may be highly beneficial for suicidal individuals, and such an intervention could be informed by this new framework. Once this immediate crisis has been addressed, other more complex theoretical approaches involving risk factors such as perceived burdensomeness could be applied in the longer term. Furthermore, our theoretical approach provides a clearer treatment target which may underlie risk factors such as entrapment and hopelessness, thereby addressing the mechanism underlying suicidal behavior more directly in therapy. This may also enable therapists to use a more client-centered and flexible approach, which could be more suitable for adaptation to challenging settings. Method of Levels (MOL) (Carey, 2006), a therapeutic application of our theoretical approach, shows evidence of promise across mental health settings (Tai, 2009, 2016; Bird et al., 2013; Carey et al., 2013; Griffiths et al., 2019a,b; Grzegrzolka et al., 2019). Our claims about the theoretical approach will be discussed in detail in the narrative review in Sections, “Predisposing to a Crisis”, “Precipitating a Crisis”, and “Mediating Suicide Behaviors”, and a detailed section on the implications for psychological interventions will be provided in Section “Clinical Implications” of the article.

The purpose of introducing the new theoretical framework is to set the stage for a new program of research which aims to test its hypotheses. For the purpose of this article we provide a narrative overview of previous theoretical literature as opposed to an exhaustive review of all theories of suicide (more extensive reviews of theoretical suicide literature can be found elsewhere, Barzilay and Apter, 2014; Gunn and Lester, 2014; Klonsky et al., 2018; Millner et al., 2020). The overview will evaluate theories that follow an ideation-to-action framework, since this framework is recommended for new models of suicide (Klonsky and May, 2014; May and Klonsky, 2016). In addition, it will include existing theories which are most consistent with our theoretical approach. Since the focus of this article is on understanding and explaining suicide from a psychological perspective, only brief reference will be made to existing treatments and/or risk assessments. Throughout the article, our definition of a suicidal crisis is consistent with the original definition provided by Hendin et al. (2007), i.e., “a time-limited psychological state that signifies acute danger of suicide,” which can occur as close as minutes before an attempt (Deisenhammer et al., 2009). We agree with previous literature that it involves intense affect (Hendin et al., 2001, 2007) which is elevated from the individual’s baseline level of affect, and involves suicidal ideation and behaviors that indicate an intent to end one’s life (Wenzel and Beck, 2008).

## What is Suicide?

In summarizing how existing theoretical models have contributed to current understanding of suicide, it is necessary to consider the key psychological processes which may occur during suicide. This includes psychological processes that may occur before an individual becomes suicidal, while they are contemplating suicide, and immediately prior to a suicide attempt. A large number of risk factors have been identified that predispose individuals to becoming suicidal through various mechanisms (O’Connor and Nock, 2014). For example, social risk factors include family history of suicide, whereas others are emotional, such as depression, or cognitive, such as experiences of hopelessness (O’Connor and Nock, 2014). Regardless of which risk factors are present, for many individuals who consider suicide, it is a response to physical or psychological pain and distress, accompanied with an unmet need to escape (Williams, 2014; Calati et al., 2015; Verrocchio et al., 2016).

When a suicidal individual is in crisis, intent to die and a motivation for ending their life act as precipitating mechanisms (Silverman et al., 2007; May and Klonsky, 2013). Individuals who both contemplate suicide (ideators) and make a suicide attempt (attempters) can experience ambivalence about suicide, if they have motivations for suicide and reasons for staying alive (Bryan et al., 2016). In addition, individuals who are in crisis can experience imagery related to suicide, such as images of desired outcomes or unwanted consequences (Hales et al., 2011).

Only a third of people who contemplate suicide make an attempt (Nock et al., 2008), which suggests that there may be critical differences between ideators and attempters (Klonsky and May, 2014). Ideators and attempters have been found to differ in terms of environmental, social, and physiological factors, such as sensitivity to pain and access to means of suicide (Klonsky and May, 2015). In addition, there is evidence that ideators and attempters differ in terms of the psychological processes underlying their experiences leading up to and during suicidal crises. For example, attempters demonstrate an increased focus on suicide-related stimuli (Cha et al., 2010) and reduced fear of death (Smith et al., 2016), relative to ideators. An individual’s suicide risk can fluctuate over a period of days or even hours (Bryan et al., 2016), and the period of time between considering suicide and making an attempt can be as short as ten minutes (Deisenhammer et al., 2009). It may be, therefore, that these fluctuations in suicide risk and rapid transitions from contemplating suicide to making an attempt are due to psychological processes, which might be different for ideators and attempters (Rudd, 2006; Bryan et al., 2016, 2019).

The new theoretical framework for understanding suicide will be introduced in the following sections. Perceptual Control Theory (Powers, 1973), the transdiagnostic framework guiding our theoretical approach, will be described. An overview will then be provided of how theoretical models have contributed to our understanding of the psychological processes which may occur during each stage of the progression from psychological distress to a suicide attempt, from distal to proximal processes. These will be grouped into the following three main headings: predisposing to a crisis, precipitating a crisis, and mediating suicide behaviors. The first heading, predisposing to a crisis, refers to psychological processes which increase individuals’ vulnerability toward experiencing a mental health problem and potentially becoming suicidal. The second heading, precipitating a crisis, refers to psychological processes which are instrumental in triggering and exacerbating a suicidal crisis. The third heading, mediating suicide behaviors, refers to psychological processes which lead an individual to attempt suicide during a suicidal crisis. For each of these headings, we will explain ways in which the new theoretical framework could potentially address unanswered questions in the existing theoretical literature. A summary of the key elements used in Sections “Predisposing to a Crisis”, “Precipitating a Crisis”, and “Mediating Suicide Behaviors” is provided in Table 1.

**TABLE 1:** Key elements of suicide which are described in the current article.

**Heading**		
**Predisposing to a crisis**	**Precipitating a crisis**	**Mediating suicide behaviors**
Cognitive-affective states which increase suicidality	Motivations and direct drivers for suicide	Capability for suicide and access to means
Responses to stressful life events and negative emotions	Ambivalence or internal conflict about suicide	Narrowing of attention
Factors which increase dispositional vulnerability	Suicide imagery	Differences in ability to imagine consequences and make decisions

## A Framework for Understanding Suicide Informed by Perceptual Control Theory

We present a new framework for understanding suicide, which guides our current and future research, including qualitative and quantitative focus on people with lived experience of suicide attempts. The new framework is informed by the principles of Perceptual Control Theory (PCT) (Powers, 1973), a transdiagnostic framework for understanding psychological well-being and distress (Mansell et al., 2012; Alsawy et al., 2014). PCT has already been applied with good effect to various areas of mental health, including psychosis, bipolar disorder, and phobias (Mansell, 2007; Mansell et al., 2014; Healey et al., 2017; Morris et al., 2018; Griffiths et al., 2019a,b). Studies on these mental health problems have supported hypotheses guided by PCT, that loss of control and goal conflict increase individuals’ distress. These theoretical constructs will be explained in the following paragraphs. Since suicide is a transdiagnostic problem, PCT may provide useful contributions to the current understanding of suicide. Explanations of terms used by the framework are provided in Table 2.

**TABLE 2:** Definition of terms (adapted from Powers, 1973; Mansell, 2005).

**Term**	**Definition**
Reference value	A “just right” state in which no action is required. This is an internal standard which is set by genetic disposition or past experience. A reference value can also be described as a goal, personal value, ideal, or principle.
Control system	A homeostatic system which acts to maintain the perception of a particular reference value.
Goal hierarchy	The structure in which personal goals are organized, ranging from abstract higher-level goals to more concrete lower-level goals.
Conflict	The experience of wanting to achieve two incompatible goals at the same time. This results from two control systems with two different reference values attempting to control the same perception. Unresolved conflict can lead to psychological distress.
Error	The discrepancy experienced when one’s current experiences do not match the way they want that experience to be (their reference value for that experience).
Awareness	The focus of an individual’s attention. An individual’s awareness moves around their goal hierarchy, although it is possible to have awareness of more than one goal at the same time.
Limited awareness	A state in which an individual is only aware of one of their goals, and is unaware of how the striving for and achievement of that goal would affect their other goals.
Awareness of the impact of suicide on one’s goals	Awareness of how one’s other higher-level goals would be adversely affected by suicide.

### People as Controllers

From a PCT perspective, all behavior, including suicidal behavior, is an attempt to act on the environment in order to achieve and maintain one’s desired experiences (perceptual states) (Powers, 1973, 1998). This process can be described as a dynamic process of control whereby the perceived effects of one’s own actions are monitored and adjusted, based on perceptual input, in a negative feedback loop (Powers, 1973). The control of these perceived effects enables an individual to match their perceptual input to a desired *reference value*, or “just right” state, and can be carried out automatically without the need for conscious awareness (Carey et al., 2015). Due to this negative feedback loop, in contrast with theories which view human experiences as resulting from cause and effect on a linear pathway, PCT views individuals as controllers of their experiences and environment (Powers, 1973; Carey, 2018).

The “just right” states that individuals control, which could also be termed as goals, values, principles, or ideals, are structured in a hierarchy ranging from higher-level goals to lower-level goals (Powers, 1973, 1998; Mansell et al., 2015). Throughout this article, in line with the PCT definition, the term *goals* includes values, principles, self-concepts, and ideals, and also refers to the concept of shared systems that an individual belongs to, such as their family, school, country, and community (Powers, 1973). Higher-level goals represent more abstract perceptions, whereas lower-level goals, further down the hierarchy, involve controlling more concrete sensory perceptions (Powers, 1973, 2005; Mansell et al., 2015). For example, a suicidal individual may have a higher-level goal to experience a sense of peace away from their current psychological distress. If they have developed a plan to attempt suicide, they may also have a corresponding lower-level goal specifying a method. Whether goals are considered to be values, principles, self-concepts, ideals, or shared systems depends on the level of the hierarchy where they are situated (Powers, 1973). However, regardless of a goal’s level or the term used to describe it, it is the *reference value* for the state that the individual would like to experience. The individual controls their perceptual input to reduce the discrepancy between their current experience and this reference value (Powers, 1973). This dynamic process of control is explained in greater detail in other literature (Powers, 1973, 1998, 2005; Mansell, 2005, 2015; Morris et al., 2016). In order to illustrate our framework, we apply it to the anonymized clinical example of Lucy.

**FIGURE 1:**
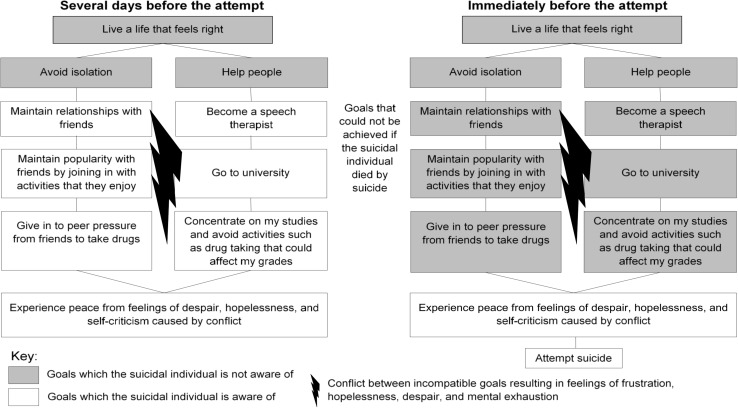
Awareness of goals at different times before a suicide attempt. This example has been adapted from clinical case material.

Lucy was a 17-year-old British female who was in her final year of school when she attempted suicide. She had struggled with mental health issues over the years following a difficult, traumatic childhood and had a history of close family members experiencing severe mental health problems. Her background and circumstances were predisposing factors for suicidality since they increased her vulnerability toward experiencing psychological distress. Before her attempt, Lucy had had aspirations to go to university and fulfill her ambition of becoming a speech therapist. Her group of friends in school were also very important to her since she found it difficult to make new friends. However, these friends began to take drugs, and pressured Lucy into joining in. Lucy experienced intense anxiety from worrying that taking drugs would affect her studies and her ability to achieve her ambitions, but also worrying that if she did not join in, her friends would reject her and she would become isolated. Lucy experienced feelings of hopelessness and despair for several months, since she could not find a solution which would enable her to achieve her ambitions without being rejected by her friends. The possibility of rejection by her friends and difficulties with fitting in with their group may have led to feelings of thwarted belongingness or perceived burdensomeness. These feelings were a consequence of conflict between Lucy’s goals (i.e., the underlying problem) preventing her from achieving either of those goals. When Lucy’s feelings of hopelessness and despair became unbearable, they acted as precipitating processes and she attempted suicide. Lucy’s experience will be referred to throughout the rest of the article and is illustrated in Figure 1.

### Reduced Control of Experiences Increases Psychological Distress

People have an intrinsic need to control their perceptions of themselves and the world (Powers, 1973; Grawe, 2006), such as maintaining a sense of safety and relationships with others (Mansell et al., 2015), and this control gives individuals a sense of purpose (Mansell and Carey, 2009). Normal human functioning is described as a state when individuals have as much control as they would like over the experiences that are most important to them (Carey, 2006). Powers (2005) states that when individuals do not have sufficient control over their experiences, they experience *error*, which is defined as a sense of discrepancy between their current experiences and the experiences they would like to have. This sense of discrepancy can result in psychological distress that may manifest in various ways, depending on the individual’s goals (Mansell et al., 2012). For example, individuals with spider phobia prefer to maintain a certain distance between themselves and any spiders, and if a spider comes within this preferred distance, they move away (Healey et al., 2017). If they are unable to move away, either due to environmental circumstances, or because they have reasons not to move away (e.g., wanting to appear capable of facing their fears), they experience psychological distress (Powers, 1973; Healey et al., 2017).

Existing literature has highlighted numerous other examples of psychological distress which result from difficulties in controlling one’s experiences. For example, individuals may experience negative thoughts or emotions as uncontrollable, or experience difficulties in controlling impulses to gamble or engage in binge eating (Tull et al., 2007; Ehring and Watkins, 2008; Fernández-Aranda et al., 2008). Furthermore, since control is so integral to individuals, in many people without mental health problems, there are times when their higher-level goals over-ride the importance of the intrinsic goals people are born with (Powers, 1973; Mansell et al., 2012). For example, individuals may have an intrinsic goal to avoid experiencing pain, but athletes participating in certain sports, such as marathon runners, willingly endure pain in order to achieve their goals, such as completing a marathon (Powers, 1973; Masters and Ogles, 2008).

Certain circumstances can affect an individual’s ability to control their experiences, such as natural disasters, or if the individual develops a serious illness, and this loss of control is often distressing (Powers, 2005; Mansell and Carey, 2009; Mansell et al., 2012). PCT proposes that there is also a more subtle way in which an individual’s control over their experiences can be reduced, which is when they try to control two incompatible experiences simultaneously (Powers, 1973). This results in conflict between the individual’s goals, which is often outside of their awareness (Carey, 2006). As a result, this conflict can remain unresolved and consequently, the individual is unable to achieve control over either goal, which may lead to ongoing psychological distress (Powers, 1973; Kelly et al., 2011b, 2015; Gray et al., 2017). *Goal conflict* can occur at times in an individual’s life when either their goals change, or their life circumstances change so that their goals are no longer compatible (Powers, 1973; Mansell et al., 2012; Carey et al., 2015). For example, if an individual becomes a parent, the newly developed goal to look after the child could cause conflict if they were also very focused on achieving their career goals and had limited time.

According to PCT, any areas of discrepancy between a current and desired experience automatically attracts an individual’s attention (Powers, 1973). By focusing on the discrepancy (problem), they may gain new insights into ways of resolving it through a process known as reorganization (Mansell et al., 2012). Reorganization describes the way in which individuals develop new ways of achieving their goals through trial and error (Powers, 1973; Carey, 2006; Mansell et al., 2012). However, if goal conflict remains outside of an individual’s awareness, and not resolved, it can become chronic goal conflict (Carey, 2006).

In the clinical example of Lucy, she experienced chronic goal conflict, which led to her attempting suicide. Specifically, she experienced peer pressure from her friends to take drugs and perceived that she would be rejected by them and become isolated if she did not join in. She also believed that taking drugs would affect her studies and her ability to get into university which, from her perspective, would mean that she is a failure. It was important for her to resolve the conflict between wanting friendships (avoiding rejection and isolation) and wanting to pursue her studies (avoiding being a failure), in order to reach satisfaction with her life. She described the process of constantly trying to find a solution to the conflict as agony, which was when the idea of suicide occurred to her as a means of ending this “agony.”

## Predisposing to a Crisis

This section will discuss psychological processes which may increase an individual’s likelihood of contemplating suicide, including psychological risk factors and ways in which individuals respond to distressing emotions. It will then explain how the new theoretical framework can be used to explain these psychological processes. Our definition of processes which predispose individuals to suicide is in line with the definition of predisposing factors used in case formulations in clinical practice (Macneil et al., 2012). This refers to any processes which may increase the individual’s vulnerability toward developing a mental health problem that may eventually result in suicide.

### Cognitive-Affective Factors Which Increase Suicidality

Previous theoretical models of suicide have highlighted the importance of specific psychological states in increasing suicidality, such as defeat, entrapment, hopelessness, perceptions of burdensomeness, thwarted belongingness, and pain, e.g. (Williams and Williams, 1997; Joiner, 2005; Johnson et al., 2008; Klonsky and May, 2015; O’Connor and Kirtley, 2018). This has provided valuable insight into the kinds of psychological distress that predispose individuals toward experiencing a suicidal crisis. Many of these theoretical models have hypothesized that the simultaneous experience of some of these cognitive-affective states increases an individual’s suicide risk. Indeed, when individuals experience some of these states simultaneously, such as defeat and entrapment, or pain and hopelessness, there is an accumulative effect, and they are more likely to contemplate suicide (Dhingra et al., 2015; Klonsky and May, 2015). However, currently known risk factors and cognitive-affective states are inaccurate at predicting suicide attempts and often result in false positive predictions (Franklin et al., 2017). Furthermore, the interactions between large numbers of risk factors and/or cognitive-affective states are difficult to comprehend due to the complexity of these interactions, and therefore do not provide a clear target for intervention (May and Klonsky, 2016; Franklin, 2019). Conversely, when interactions between small numbers of risk factors are analyzed, they have low explanatory power in understanding suicide attempts (Franklin, 2019).

Recent theoretical suicide literature has proposed that since the interplay between risk factors for suicide is so complex, it may be more useful to understand suicide in terms of psychological primitives (Franklin, 2019). Psychological primitives are defined as the “fundamental (i.e., do not rely on anything else psychological to exist) and irreducible (i.e., cannot be reduced to anything else psychological) psychological entities from which all psychological phenomena emerge” (Ortony and Turner, 1990; Russell, 2003; Barrett, 2009, 2012; Barrett and Satpute, 2013; Franklin, 2019). More specifically, according to Franklin (Franklin, 2019), psychological phenomena emerge when individuals interpret internal stimuli (e.g., their experience of affect) and external stimuli using their conceptual knowledge. Franklin (2019) suggested that explaining suicide in terms of this kind of core psychological process may be more beneficial than investigating complex interactions between risk factors. Indeed, progress has been made in understanding the pathways to mental health problems and reduced well-being using similar approaches, such as examining psychological processes which determine response to various factors, such as past experiences of trauma and socio-economic status (Kinderman et al., 2015). Therefore, an approach which focuses on basic psychological processes may provide a more in-depth understanding of a common pathway to suicide attempts than investigating the interplay between specific risk factors. One potential area for refinement would be to identify common psychological mechanisms, by which interactions between these cognitive-affective states (e.g., perceived burdensomeness and thwarted belongingness), might increase and maintain individuals’ psychological distress, thereby increasing their likelihood of contemplating suicide.

#### The PCT Concept of Chronic Goal Conflict Integrates Risk Factors From the Existing Literature

Our new theoretical framework has potential to address the unanswered questions in existing literature which we have highlighted so far by providing an explanation of basic psychological processes that maintain distress. Our framework integrates and potentially explains states such as thwarted belongingness, psychological pain, perceived burdensomeness, hopelessness, defeat, and entrapment, which are predicted to be instrumental in suicide by other recent theories (Joiner, 2005; Johnson et al., 2008; Klonsky and May, 2015; O’Connor and Kirtley, 2018), as arising due to goal conflict. It is clear that these states of mind, such as hopelessness and entrapment, commonly have a crucial role in suicide (Hendin et al., 2007; De Beurs et al., 2019). However, from a PCT perspective, these emotional states are all examples of the *result* of chronic unresolved conflict between goals, and it is the chronic unresolved conflict which is the *mechanism* underlying the distress (Carey, 2006). For example, it is likely that a sense of entrapment is experienced when an individual has attempted many different solutions to resolve a particular conflict, none of which have been successful (Gilbert and Allan, 1998). This conflict between goals, which remains unresolved, is likely to lead to the sense of entrapment, whilst having many unsuccessful attempts at resolving the problem is likely to accompany a sense of defeat, i.e., the failed struggle (Gilbert and Allan, 1998; Taylor et al., 2010). This is consistent with evidence that internal entrapment, defined as a sense of being trapped by one’s own thoughts and feelings (Gilbert and Allan, 1998), is more strongly associated with suicidal ideation than external entrapment, defined as being trapped by external circumstances (Gilbert and Allan, 1998; Rasmussen et al., 2010; Owen et al., 2018; De Beurs et al., 2019).

Furthermore, there is growing evidence that defeat and entrapment both reflect a transdiagnostic psychological mechanism which underlies suicidal ideation (Griffiths et al., 2015; Owen et al., 2018), and we suggest that this underlying mechanism is goal conflict. In the case of perceived burdensomeness, if one of an individual’s higher-level goals involves trying not to be a burden to other people, they will take steps to maintain this. However, they may also have reasons to avoid taking these steps, such as if they need social support in order to manage the distress arising from a traumatic life event or symptoms of a mental health problem. These reasons for both avoiding being a burden to others and seeking help or support from others will entail conflict between the two incompatible goals. As a result of this conflict, neither “just right” state is achieved (Powers, 1973; Carey, 2006), and so the individual is unable to adequately control their experience of not being a burden. If such a conflict remains unresolved, the individual will experience chronic loss of control of this important higher-level goal, which is likely to cause psychological distress (Mansell et al., 2012). Therefore, the emotional states that play key roles in other models are integrated and explained as arising due to conflict between important life goals, and the way in which this conflict is experienced depends on individual circumstances (Carey, 2006; Mansell et al., 2012, 2015; Mansell and McEvoy, 2017). Indeed, there is evidence that goal conflict underlies psychological distress and reduced wellbeing (Kelly et al., 2011a, 2015; Gray et al., 2017).

Moreover, we posit that the reason the *combination* of thwarted belongingness and perceived burdensomeness leads so many individuals to consider suicide (Joiner, 2005) is due to goal conflict. The conflict most likely includes a goal to not feel like a burden to other people and a goal to feel a sense of belongingness, both of which are likely to come from a more general higher-level goal to connect to other people, which may be further up in the goal hierarchy (Mansell et al., 2015). If an individual feels that they may be a burden to other people, the steps they take to reduce their perceived burdensomeness may also decrease their sense of belongingness, resulting in feelings of thwarted belongingness. If they take steps to increase their sense of belongingness, such as relying on other people for social support, they may begin to perceive themselves as being more of a burden, thereby increasing their feelings of perceived burdensomeness. The chronic unresolved conflict which, for that individual, may arise from incompatibility between these goals (Carey, 2006) may lead to the kind of ongoing psychological distress that causes some individuals to consider suicide. We agree with theories which postulate that psychological pain plays an important role in suicide (Klonsky and May, 2015), but we posit that the mechanism underlying the psychological pain is chronic goal conflict. Therapeutic applications of this theoretical approach to understanding psychological distress would aim to target the underlying mechanism, i.e., goal conflict, rather than the array of outcomes that are generated by it, such as entrapment or hopelessness. We posit that if goal conflict is the key target for intervention in therapy, psychological states such as feelings of entrapment, hopelessness, or perceived burdensomeness will reduce; whereas targeting these states (e.g., perceived burdensomeness) in therapy addresses the problem less directly. From this perspective, suicidal individuals’ problems may be resolved less efficiently or may not be fully resolved, and they may be more likely to continue experiencing suicidal thoughts than if the goal conflict was addressed directly.

### Responses to Stressful Life Events and Negative Emotions

Cognitive behavioral models of suicide posit that the way in which individuals respond to stressful life events and negative emotions can increase their likelihood of experiencing suicidal crises, such as rumination over negative experiences or feelings of thwarted belongingness (Johnson et al., 2008; Wenzel and Beck, 2008; Williams et al., 2016). There is evidence that responses such as rumination, experiential avoidance, or avoidance-based coping attempts, are associated with increased distress (Penley et al., 2002; O’Connor et al., 2007; Karekla and Panayiotou, 2011). A large study involving 32,827 participants from the general population demonstrated that these kinds of psychological processes mediate the impact of life events, risk from familial mental health history, and social circumstances, such as income, on mental health (Kinderman et al., 2013). Since none of these psychological processes reliably differentiate between ideators and attempters (May and Klonsky, 2016; Franklin et al., 2017), they may increase some individuals’ vulnerability towards experiencing mental health problems, but are unlikely to be involved in the final common pathway to suicide attempts.

#### Responses to Stressful Life Events and Negative Emotions From a PCT Perspective

Our theoretical framework has potential to explain how psychological processes such as rumination and experiential avoidance can exacerbate distress which may cause an individual to consider suicide. From a PCT perspective, the process of reorganization is necessary to resolve goal conflict, but for this process to occur, the individual needs to focus their awareness on the level above the conflicting goals within their goal hierarchy (Carey, 2006). Awareness of higher-level goals which are above the level of the goal conflict is necessary to enable the individual to identify the underlying purposes of the conflicting goals, and consequently re-evaluate new ways of achieving those goals (Mansell et al., 2012; Carey et al., 2014a). For example, a case study (Grzegrzolka and Mansell, 2019) described a client who was in conflict, but who experienced therapeutic change by focusing his awareness on the level above the conflicting goals. The client wanted to change his principles of overworking and socializing by frequently drinking at the pub, in order to feel less tired throughout the week. He was in conflict since he did not want to change these principles, since he values his work and his social life. Therapy helped him to focus his awareness above the level of these conflicting principles, and he had a realization regarding his own identity which appeared to be at higher level. This realization was that being a mental health professional himself, he should be willing to make changes necessary for his own well-being. This realization enabled him to make changes to his principles, such as how much he prioritized his work or social life, so that they were no longer in conflict with each other.

However, if the individual only focuses their attention on the level of the two conflicting goals, they may not reach a solution to their problem (Carey, 2006; Mansell et al., 2012; Carey et al., 2015), and the resulting distress may increase their risk of suicide. This exclusive focus on the level of the conflicting goals and subsequent distress is reflected in the descriptions of various transdiagnostic strategies which have been examined in other literature (Williams et al., 2016; Mansell and McEvoy, 2017). These strategies include thought suppression, worrying, self-punishment, experiential avoidance, and substance misuse, and are used as a means of controlling one’s experiences (Carver et al., 1989; Hayes et al., 1996; Purdon and Clark, 1999; Moore and Abramowitz, 2007). However, since many of these strategies are associated with increased distress (Hayes et al., 1996; Purdon and Clark, 1999; Penley et al., 2002; Moore and Abramowitz, 2007; Karekla and Panayiotou, 2011) and do not address the underlying goal conflict, they exacerbate the problem further and the individual remains in chronic conflict (Mansell, 2011a). For example, instead of talking through her problems with family members or friends, Lucy tried to suppress thoughts that she might fail at her exams whenever they came into her awareness.

### Factors Which Increase Dispositional Vulnerability

While the primary focus of this section is on psychological processes that increase individuals’ likelihood of contemplating suicide, the role of dispositional vulnerability factors such as genetics, biological circumstances, and an individual’s personality and psychological characteristics must be acknowledged (Wenzel and Beck, 2008; Brodsky and Mann, 2017). The Stress-diathesis model of suicide (Brodsky and Mann, 2017) posits that trait factors such as lower serotonin function, genetics, and the impact of childhood experiences increase individuals’ vulnerability towards suicide. When these trait factors interact with state factors, such as symptoms of psychosis, or negative life events such as losing one’s job, individuals may experience suicidal ideation which could lead a suicide attempt (Brodsky and Mann, 2017). Wenzel and Beck’s cognitive model of suicidal behavior (Wenzel and Beck, 2008) holds similar assumptions but from a cognitive perspective. It proposes that suicidal individuals have psychological characteristics, such as overgeneral memory, problem-solving deficits, maladaptive cognitive styles, and personality traits such as neuroticism, which predispose these individuals to becoming suicidal in the presence of severe life stressors (Wenzel and Beck, 2008).

#### A PCT Understanding of Dispositional Traits

From our theoretical perspective, all dispositional factors which increase vulnerability to mental health problems are integrated using the concepts of control and conflict (Carey et al., 2014a,b; Mansell et al., 2015; Mansell, 2016). According to PCT, genetic traits, biological circumstances, psychological characteristics, and environmental factors impact on individuals’ ability to control aspects of their lives which are important to them, potentially leading to poorer wellbeing and mental health (Mansell et al., 2012, 2015; Carey et al., 2014a,b; McEvoy et al., 2016). For example, Alzheimer’s disease, which occurs partly due to genetic factors (Moreno-Gonzalez et al., 2020), can negatively impact on individuals’ mental health and lead to suicide in some cases (Purandare et al., 2009; Seyfried et al., 2011; Cui et al., 2019). The disease affects individuals’ ability to maintain their sense of self and function in their daily lives, by reducing their ability to concentrate, communicate, and complete tasks such as making tea (Bastin et al., 2010; McEvoy et al., 2016). This lack of control over their lives can lead to psychological distress and reduced wellbeing (McEvoy et al., 2016). Similarly, some individuals have personality traits that predispose them towards hypomanic experiences, which in some cases lead to a diagnosis of bipolar disorder, a risk factor for suicide (Kwapil et al., 2000; Brodsky and Mann, 2017). From a PCT perspective, it is not the hypomanic experiences which directly cause mental health problems, but resulting chronic conflict between important life goals of these individuals (Mansell, 2016). Lastly, PCT posits that environmental factors, such as experiences of trauma, impact on mental health when they result in chronic conflict between an individual’s goals, thereby reducing their control over their experiences (Carey et al., 2014b). One example of goal conflict underlying trauma is someone wanting to forget their experiences of abuse so that they can move on, but wanting to keep remembering their abuser in order to continue hating them (Carey et al., 2014b). Our theoretical framework posits that all dispositional trait and state factors, such as biological, environmental, psychological, and neurocognitive factors (Wenzel and Beck, 2008; Brodsky and Mann, 2017), can increase vulnerability towards suicide by reducing individuals’ control over their experiences.

The theoretical framework we present, as applied to suicide, has important advantages over theoretical models such as the Stress-diathesis model of suicide (Brodsky and Mann, 2017) and a cognitive model of suicidal behavior (Wenzel and Beck, 2008). Firstly, as previously mentioned, it offers an explanation of core psychological processes which underlie all dispositional trait and state factors, therefore providing an account which does not rely on analyses of interactions between risk factors. Secondly, both of these models assume linear cause and effect, but there is evidence that suicide risk fluctuates and does not necessarily follow a linear pathway (Bryan et al., 2016, 2019; Klonsky et al., 2018). In contrast, our theoretical framework, by understanding human functioning as a negative-feedback process by which individuals continually attempt to control their experiences (Carey et al., 2014b), provides a more dynamic way of understanding fluctuations in suicide risk. Thirdly, these models describe fewer details on how individuals move from suicidal ideation to making an attempt, whereas our theoretical framework provides an in-depth explanation of psychological mechanisms by which individuals attempt suicide. The latter two points will be explained in greater detail in the “Precipitating a Crisis” and “Mediating Suicide Behaviors” sections.

## Precipitating a Crisis

This section will describe psychological processes which may trigger a suicidal crisis, including individuals’ motivations for suicide. The potential roles of ambivalence about suicide and suicide imagery before and during suicidal crises will also be considered. Our definition of processes which precipitate a crisis is consistent with definitions of precipitating factors within the literature on clinical case formulations (Macneil et al., 2012), i.e., processes which precede the onset of a suicidal crisis. We consider processes which predispose individuals to suicide to be distinct from precipitating processes, since the former increase an individual’s general vulnerability towards mental health problems, whereas the latter occur more specifically and immediately before the onset of a suicidal crisis.

### Motivations and Direct Drivers for Suicide

It is vital to understand suicidal individuals’ motivations for suicide, in order to address these motivations within psychological interventions (May and Klonsky, 2013). This is particularly crucial since individual circumstances can affect the influence of risk factors on suicidal behavior (Pompili, 2018), and the relationship between risk factors and suicidal behavior is complex (Pompili et al., 2010). Reviews of existing evidence indicate that certain biological risk factors and cognitive processes are only problematic in particular contexts (Carey et al., 2014b).

There have been few studies on direct drivers of suicide or motivations for suicide which have been guided by theoretical models (May and Klonsky, 2013). Direct drivers of suicide are defined as the thoughts, feelings, and behaviors which lead to the individual becoming suicidal, but which are more specific to the person’s individual circumstances than emotional states, such as hopelessness or entrapment (Jobes, 2006; Tucker et al., 2015). They include an individual’s internal experiences and what makes specific emotional states, such as thwarted belongingness, problematic for them (Tucker et al., 2015). Tucker and colleagues (Tucker et al., 2015) give the example of a man who wants to end his life because he perceives himself to be a burden to others (the direct driver), which are due to his inability to keep a job, bad financial circumstances, and mental health problems (indirect drivers). An increased theoretical focus on drivers or motivations for suicide may be helpful in future research, particularly given the limitations of risk factors in providing a greater understanding of suicide (Franklin et al., 2017). Tucker and colleagues emphasized the importance of understanding the idiosyncratic direct driver for each suicidal individual, whether that driver is perceived burdensomeness, impulsivity, or interpersonal isolation (Ellis et al., 2012; Tucker et al., 2015), such as what makes a particular individual feel like a burden. However, a theoretical framework hypothesizing a common psychological process that motivates individuals to consider suicide, regardless of their idiosyncratic experiences and individual drivers, may provide a clearer target for psychological interventions. It may also provide insight beyond what is currently known from research on emotional states or risk factors.

#### Loss of Control Precipitates Suicidal Crises

The new theoretical framework for understanding suicide offers the explanation that loss of control is the common psychological process underlying motivations for suicide regardless of individuals’ idiosyncratic experiences. We posit that if goal conflict remains unresolved, individuals may experience an acute loss of control as a result of neither goal being achieved (Mansell, 2005), potentially resulting in sufficient psychological distress for the individual to experience a suicidal crisis. This is consistent with previous theoretical claims that individuals experience the greatest distress when they perceive a discrepancy between their perception of their current experiences and the states they would like to experience (Williams et al., 2016).

However, there are subtle differences in the way in which our account conceptualizes these ideas. Some accounts postulate that distressing emotions trigger a sense of discrepancy (Williams et al., 2016). In contrast, we posit that anyone who is unable to control the experiences which are most important to them will experience a prolonged sense of discrepancy or loss of control (Carey, 2006). We posit that the distressing emotions arise when individuals become aware of this discrepancy, and that the emotional state an individual experiences will depend on which kinds of goals they are unable to achieve (Mansell et al., 2015). For example, an individual who feels unable to achieve their goal of feeling a sense of belonging with other people may experience feelings of thwarted belongingness.

If the use of strategies to cope with this loss of control, such as taking drugs, drinking alcohol, or engaging in experiential avoidance, reduce an individual’s distress on a short-term basis, they may continue using these strategies (Mansell and McEvoy, 2017). However, since these strategies prevent the individual’s awareness from focusing on the level of the goal hierarchy where it needs to be, i.e., the level above the conflicting goals, reorganization cannot take place (Mansell et al., 2012, 2015; Mansell and McEvoy, 2017). Therefore, the continued sense of loss of control arising from the conflicting goals (Mansell et al., 2012) may result in individuals becoming more likely to experience a suicidal crisis. This loss of control can involve an individual feeling unable to make decisions, not knowing what they want, or experiencing a loss of sense of identity, depending on which level of the goal hierarchy the loss of control occurs (De Hullu, 2020). For example, when Lucy initially suppressed thoughts that she might fail at her exams, this redirected her attention away from the goal conflict, and she felt less distressed temporarily. However, the more she suppressed these thoughts, the more often they returned to her awareness, making her experience an even greater sense of loss of control. This led to a chronic and overwhelming sense of loss of control and as a result, she began to frequently experience severe psychological distress, which eventually led to a suicidal crisis.

#### Suicide as a Means of Controlling Perceptual Experiences

According to our framework, individuals’ motivations for suicide occur as a result of having a specific higher-level goal which they believe could be achieved by suicide. For example, Lucy believed that the distressing emotions she was experiencing, including a feeling of “agony,” would end if she died by suicide. We postulate that in many cases these higher-level goals involve ending or escaping from physical or psychological pain, but that this is not necessarily the case.

Since control is a dynamic process (Powers, 1973), this may explain fluctuations in suicide risk (Bryan et al., 2016); it is posited that individuals’ desire to attempt suicide at any given moment depends on their control over goals which could be achieved by suicide. For example, during moments when Lucy experienced less distress, she was less motivated to attempt suicide. This dynamic process of control is explained and illustrated in other literature (Powers, 1973, 2005; Mansell and Huddy, 2018), including mental health literature (Mansell, 2005; Morris et al., 2016), and from 3.00 min onward in a video explaining PCT (Mansell, 2011b).

### Ambivalence or Internal Conflict About Suicide

Feelings of ambivalence or internal conflict are common in the build up to and during suicidal crises. In a sample of 888 attempters, 85.4% experienced ambivalence about whether they wanted to die (Kim et al., 2018). Clinicians are advised to explore ambivalence about suicide (Berman and Silverman, 2014) in assessments such as the Collaborative Assessment and Management of Suicidality (CAMS) (Jobes, 2006). Furthermore, ambivalence towards suicide plays a crucial role, on an ongoing basis, for multiple suicide attempters, both causing them distress and keeping them alive (Bergmans et al., 2017). Investigating psychological processes involved in ambivalence about suicide may lead to a greater understanding of the mechanisms underlying suicide attempts, particularly since ambivalence can deter individuals from attempting suicide (Jobes, 2006; Bryan et al., 2016).

The role of ambivalence about suicide has been acknowledged by some suicide theorists (Shneidman, 1964; Stengel, 1964; Linehan et al., 1983; Jobes, 2006) and theoretical models, such as the Fluid Vulnerability Theory (FVT) (Rudd, 2006) of suicide. However, a limitation of existing literature is that few theoretical models which follow an ideation-to-action framework include ambivalence about suicide as a key concept, despite its important role in suicide (Jobes, 2006; Bryan et al., 2016). The FVT is the only model following an ideation-to-action framework which fully considers the role of ambivalence (Bryan, 2020). Although the FVT offers a useful approach to understanding fluctuations in individuals’ wish to live and wish to die, it provides fewer details on the psychological processes which may underlie these fluctuations. A theoretical framework which provides a more in-depth understanding of ambivalence than previous theoretical models may be useful in guiding future research. Specifically, a theoretical framework specifying the psychological processes involved in the balance between one’s reasons for wanting to live and wanting to die in greater detail would expand on previous theoretical accounts. This could include mechanisms by which individuals’ focus on these reasons varies over time.

#### Ambivalence About Suicide Reflects Internal Conflict Between Goals

The new theoretical framework for understanding suicide expands on accounts from previous literature by providing a deeper explanation of the psychological processes involved in the balance between one’s reasons for living and reasons for wanting to die. From a PCT perspective, the reasons for living and reasons for wanting to die which are referred to in previous literature (e.g., Jobes, 2006) are conceptualized as higher-level goals. Individuals may experience ambivalence about suicide when they have awareness of the higher-level goal motivating them to end their lives but are also aware of higher-level goals or ideals which would be negatively impacted on if they died by suicide. Since both types of goal are incompatible, this is another example of goal conflict, which can become chronic if it remains unresolved (Carey, 2006; Mansell et al., 2012). We posit that ambivalence about suicide can be distressing because chronic unresolved conflict prevents individuals from having enough control over their experiences (Powers, 1973; Carey, 2006; Carey et al., 2014a).

However, ambivalence about suicide can also be protective against suicide attempts (Bergmans et al., 2017), since an awareness of goals which would be negatively impacted on by suicide may deter individuals from attempting suicide (Tarrier et al., 2013). We agree with Klonsky and May (Klonsky and May, 2015) that connectedness is a major protective factor, but we conceptualize it as the amount of control an individual has over the perceptual state of feeling connected. We posit that greater awareness of a higher-level goal to feel connected is likely to be protective against suicide. Our theoretical account is consistent with findings that ambivalence can be both distressing and protective in suicidal individuals (Bergmans et al., 2017). However, it goes beyond recent theoretical accounts of ambivalence about suicide (Linehan et al., 1983; Jobes, 2006; Rudd, 2006), since we posit that one’s combined reasons for living and reasons for dying are simultaneously associated with distress, due to the loss of control they may entail.

### Suicide Imagery

Mental imagery plays a role in suicide, particularly during suicidal crises, since it is essential for planning, goal setting, and choosing between options, and enables individuals to rehearse events in their minds (Schacter et al., 2008; Crane et al., 2012). Individuals experience mental imagery when thinking about potential consequences of their actions (Gilbert and Wilson, 2007), and imagery influences future behavior (Hales et al., 2011). Suicidal individuals can experience “flash-forward” images of their potential suicide, containing images of methods and potential desired and undesired consequences, such as family members’ reactions (Hales et al., 2011). The role of these images may be complex in suicide, since both ideators and attempters experience suicide imagery, and imagery deters some individuals from engaging in self-harm and attempting suicide (Hales et al., 2011; McEvoy et al., 2017). Therefore, experiences of suicide imagery may be linked to individuals’ reasons for wanting to die and for wanting to live, and may affect or be influenced by the balance between these reasons.

In addition, addressing the content of mental imagery in therapy can reduce psychological distress (Holmes et al., 2007a) and reduce suicidal ideation in suicide attempters (Rahnama et al., 2013). For example, the Broad Minded Affective Coping (BMAC) task (Tarrier, 2010), a key component of Cognitive Behavioral Therapy for Suicide Prevention (CBSP) (Tarrier et al., 2013), uses mental imagery to reconstruct positive memories, thereby increasing access to their associated positive emotions. Therefore, the images which suicidal individuals experience warrants further investigation which is guided by theoretical hypotheses.

Currently, models such as the Integrated Motivational-Volitional Model of suicide (IMV) (O’Connor and Kirtley, 2018) explain the role of suicide imagery as a form of cognitive rehearsal of suicide. However, this does not account for the role of imagery in cases when it deters individuals from engaging in self-harm or attempting suicide (Hales et al., 2011; McEvoy et al., 2017). A more in-depth explanation of suicide imagery is needed, which explains the mechanisms by which imagery both deters individuals from and influences individuals to attempt suicide, and how these mechanisms relate to ambivalence about suicide.

#### Suicide Imagery Reflects Individuals’ Goals

Our framework for understanding suicide offers a theoretical explanation of the mechanisms by which images of potential consequences influence whether individuals will attempt suicide, and their relationship with ambivalence about suicide. The PCT explanation of mental imagery is consistent with previous cognitive literature stating that imagery is a means of rehearsing future actions and simulating potential consequences (Gilbert and Wilson, 2007; Schacter et al., 2008). According to our framework, individuals experience “flash-forward” suicide imagery when they have a higher-level goal which could be achieved by suicide that has come into their awareness. This enables individuals to imagine how the goal of ending their life could be achieved, so this aspect of our account is consistent with theoretical accounts conceptualizing suicide imagery as a form of cognitive rehearsal for suicide (O’Connor and Kirtley, 2018). For example, prior to making a suicide attempt, Lucy imagined herself cycling in front of cars on the road as a means of ending her life.

Our account also differs from some previous theoretical accounts, such as the IMV (O’Connor and Kirtley, 2018), since our account also emphasizes a potentially protective aspect of suicide imagery. We postulate that some suicide imagery occurs due to the individual becoming aware of higher-level goals which would be negatively impacted on by suicide. Therefore, some imagery may enable individuals to become more aware of potential negative consequences of suicide which are linked to their reasons for living, and thus have a positive impact (Tarrier, 2010; Tarrier et al., 2013). For example, after Lucy had survived a suicide attempt, she experienced images of her family’s reaction if she had died, and felt acute sadness and regret that her actions could have had these consequences. This account is consistent with existing therapeutic techniques which utilize protective aspects of mental imagery in clinical practice. For example, the BMAC (Tarrier, 2010; Tarrier et al., 2013) aims to support suicidal individuals’ imaginal rehearsal of key positive memories, which are typically key social events, such as their wedding day, one of their children being born, or a family holiday.

## Mediating Suicide Behaviors

The following section will discuss psychological processes which may cause individuals to attempt suicide during a suicidal crisis.

### Capability for Suicide and Access to Means

Recent theoretical models of suicide which follow an ideation-to-action framework, including the IMV (O’Connor and Kirtley, 2018), 3-Step Theory (3ST) (Klonsky and May, 2015), and Interpersonal Theory of Suicide (IPTS) (Joiner, 2005), posit that individuals progress from suicidal ideation to suicide attempt if they have sufficient capability for suicide. Joiner (2005) argues that for evolutionary reasons, people avoid threats such as the risk of injury or death, and therefore, the act of attempting suicide involves overcoming one’s fear of death or sensitivity to pain. Through the process of habituation, individuals’ fear of death and pain sensitivity decrease, and consequently, these individuals are more capable of making a suicide attempt (Joiner, 2005).

The 3ST and IMV have expanded on these ideas by proposing that environmental and social factors, such as access to means of suicide and exposure to suicide attempts made by family members, also influence whether individuals attempt suicide (Klonsky and May, 2015; O’Connor and Kirtley, 2018). These ideas have been supported by recent empirical evidence (Klonsky and May, 2015; Smith et al., 2016). However, while knowledge of these factors is important for suicide prevention strategies on a societal level (Zalsman et al., 2016), it does not highlight a clear treatment target which is amenable to psychological interventions. Furthermore, it does not explain fluctuations in individuals’ ambivalence about attempting suicide (Bryan et al., 2016; Bergmans et al., 2017). Therefore, a further refinement to these theoretical ideas could be to specify precise psychological processes occurring during a suicidal crisis that contribute to an individual’s decision to make a suicide attempt. This would guide further research on these psychological processes, thereby informing ways in which psychological interventions could be refined to directly address such processes.

### Narrowing of Attention

Theorists have postulated that some individuals experience a narrowing of attention (or “cognitive constriction”), only focusing on certain aspects of their experiences when they are feeling suicidal (Shneidman, 1964; Johnson et al., 2008; Wenzel and Beck, 2008; Williams et al., 2016). Indeed, suicide-specific rumination, defined as a fixation on one’s suicide-related thoughts and plans (Rogers and Joiner, 2017), predicts attempts over and above other risk factors (Rogers and Joiner, 2018). In addition, individuals at high risk of suicidal behavior experience difficulties in controlling the focus of their attention, and attempters demonstrate reduced cognitive inhibition compared to ideators (Richard-Devantoy et al., 2015; Thompson and Ong, 2018). However, an explanation of these differences in focus of attention and attentional control between ideators and attempters is not included in theoretical models which fit the recommended ideation-to-action framework (Klonsky and May, 2014), such as the IMV, 3ST, and IPTS. Furthermore, no theoretical models currently exist which attempt to integrate these related but separate constructs. Therefore, further refinements to the existent theoretical literature could integrate these findings and explain how these psychological processes influence whether individuals attempt suicide.

#### Narrowing of Attention and Limited Awareness: The Common Pathway From Ideation to Suicide Attempts From a PCT Perspective

The core predictions of our framework for understanding suicide, which will now be outlined, have the potential to integrate these findings on attentional control and narrowing of attention using the concept of limited awareness. Our framework proposes that limited awareness is the final common psychological pathway underlying suicide attempts, regardless of which risk factors are present in an individual’s life. Even though the loss of control resulting from goal conflict automatically attracts an individual’s attention, for many people, their attention is naturally drawn away from any prolonged focus on the problem to other more wide-ranging priorities in their lives (Mansell et al., 2012; Kelly et al., 2013). People vary in their propensity to balance focusing on pursuing particular goals with being flexible enough to shift their attention to wider issues in their lives (Kelly et al., 2013).

*Limited awareness* occurs when individuals become focused on the pursuit of one particular goal to the extent that they lose sight of how this might impact upon other goals (Powers, 1973; Mansell, 2005). This can include a limited awareness of both concrete lower level-goals and more existential higher-level goals or values, such as goals specifying the sort of person they want to be or principles they prefer to follow. This psychological process is theorized to occur across a range of psychological difficulties (Mansell et al., 2015) and is posited to be the psychological mechanism by which individuals attempt suicide. We posit that limited awareness occurs when suicidal individuals become exclusively focused on suicide as a goal, in an attempt to avoid experiencing memories and feelings that are part of their important life goals. For example, they may have an important life goal to maintain a close relationship with their children, in which case memories of spending time with their children and the associated feelings would remind them of this goal. They may avoid experiencing these memories or feelings if they are experiencing loss of control in these areas of their lives, which could trigger overwhelming and extreme distress whenever their focus of awareness is placed upon them. Therefore, we conceptualize suicide as a means by which individuals attempt to increase their sense of control, whilst concurrently avoiding the experience of memories or reminders of their important life goals.

In reality, if an individual ends their life, it would have a negative impact on the achievement of their other goals, thus bringing about consequences which conflict with the achievement of these goals. However, our framework posits that an individual contemplating suicide can become so focused on the goal of ending their life, in order to regain control, that the consequences of suicide which conflict with other important life goals remain outside of their current awareness. This exclusive concentration upon ending one’s life occurs when suicide as a means of regaining control becomes the focus of attention, often due to the chronic error arising from lost control of other goals (Powers, 2005; Carey, 2006; Mansell et al., 2012).

The process of focusing on the goal to end one’s life and having limited awareness of other goals may explain what is referred to as “tunnel vision” in anecdotal accounts of suicide attempts (Wenzel and Beck, 2008). During this process, individuals may experience imagery of methods of suicide, enabling them to mentally simulate a suicide attempt (Hales et al., 2011), and imagery of outcomes they want to achieve by ending their life, such as an end to their suffering (Crane et al., 2012). For example, Lucy imagined being hit by a car and the overwhelming stress that she was experiencing ending very suddenly. An individual’s limited awareness makes them less likely to imagine consequences which they would prefer to avoid, such as upsetting their family. As a result, they are not deterred from attempting suicide and remain focused upon this goal.

The theoretical idea of limited awareness is consistent with findings from cognitive psychology literature that individuals have goals which they are not consciously aware of, and that these unconscious goals can conflict with each other (Bargh et al., 2001). One of these conflicting unconscious goals can become more dominant than another (Moskowitz et al., 1999; Bargh et al., 2001), thus preventing the individual from becoming aware of the less dominant goal (Moskowitz et al., 1999). Therefore, if a goal to end one’s life is more dominant than other goals, the individual is prevented from becoming aware of their other goals associated with reasons for living.

In the clinical example, after a prolonged period of despair and ambivalence about suicide, Lucy became completely focused on ending her agony and despair. Due to the “agony” she was experiencing, she found it difficult to envisage achieving any other goal unless the agony went away. She became so focused on the goal of “ending my agony” that she was no longer attentive to any of her other goals, which obviously would not be achieved if she died by suicide, despite these goals underlying her conflict. This exclusive focus upon the “ending my agony” goal also occurred since Lucy was already experiencing a loss of control and, in order to consider her other goals, she would have had to confront her fears of being rejected or being a failure more directly. Confronting these fears, and the resulting loss of control, would feel overwhelming. As such, Lucy was conscious of not wanting to perceive the feeling of intense anxiety associated with these fears, which she would have experienced if she became more aware of these goals. However, she was not fully conscious of wanting to avoid thinking about the negative consequences of suicide for these life goals. These other goals included maintaining contact with her friends and pursuing a career as a speech therapist. Limited awareness of these other goals meant that Lucy did not have to imagine any negative consequences of ending her life (e.g., no further contact with friends) and consequently attempted suicide.

#### Responses to Ambivalence About Suicide Affect Awareness of Goals

Ambivalence about suicide can be distressing (Bergmans et al., 2017), and we posit that this is due to the underlying goal conflict between one’s reasons for living and reasons for dying, since conflict leads to loss of control (Powers, 1973; Mansell et al., 2012). Individuals may avoid focusing their awareness on the conflicting goals, since memories and reminders of these goals may lead to an even greater and overwhelming loss of control. This is reflected in strategies which have been investigated in the transdiagnostic literature, such as thought suppression, experiential avoidance, or drinking alcohol to block out unwanted thoughts and feelings (Carver et al., 1989; Hayes et al., 1996; Purdon and Clark, 1999). Similarly, individuals may use appraisals which minimize their perception of the impact of suicide on their life goals, such as telling themselves that others will not miss them (Tarrier et al., 2013). We posit that these strategies are all means of avoiding placing a focus of awareness upon one’s goals which are in conflict (Mansell et al., 2015; Mansell and McEvoy, 2017). We also suggest that doing so can impede an individual’s access to potentially distressing memories or reminders of goals that may underlie their reasons for living. This prevents individuals from remaining aware of goals which would be negatively impacted by a death from suicide.

For example, an individual who is experiencing feelings of perceived burdensomeness may feel that others would be better off without them. Even if they are fully aware of how upset their family and friends might be should they take their own life, they may still feel that in the long term it is better to prioritize reducing the burden they place upon others. As a result, the individual may wish to avoid feeling any guilt associated with thoughts that their death could upset others. When they are considering suicide, they may become aware of this guilt, since we posit that individuals automatically become aware of valued goals if they are about to engage in behavior which would prevent the achievement of these goals. The individual may employ strategies such as thought suppression or drinking alcohol in order to avoid experiencing the guilt about the pain caused to their family as a result of their death. If the person frequently uses strategies to avoid distress associated with these other goals, this is likely to result in limited awareness of how their other goals (such as to avoid upsetting their family) would be negatively impacted upon by suicide.

We also posit that the limited awareness resulting from moving one’s awareness towards less distressing goals, such as by distracting oneself using alcohol, may be experienced as feelings of emotional numbing or dissociation (Holmes et al., 2005; Mansell and Carey, 2012). These theoretical ideas are consistent with evidence from existing literature that high levels of dissociation are associated with an increased number of suicide attempts, regardless of an individual’s ability to tolerate pain (Rabasco and Andover, 2020). Furthermore, emotional numbing is associated with suicidal ideation, and suicide plans and attempts among ideators (Afzali et al., 2017).

In contrast, some individuals allow themselves to experience reminders of goals that would be negatively impacted upon by their death from suicide (Hales et al., 2011; Crane et al., 2012). We posit that these individuals are more likely to maintain a greater awareness of these goals and are less likely to attempt suicide. This is consistent with psychological interventions that aim to increase suicidal individuals’ awareness of their reasons for living. For example, clinicians using the Cognitive Behavioral Prevention of Suicide (CBPS) (Tarrier et al., 2013) explore the meaning and emotions associated with the client’s negative beliefs about suicide. This has the aim of encouraging ambivalence and ensuring that the client maintains a full awareness of the negative impact of suicide on their other goals, especially when the client is in crisis (Tarrier et al., 2013). Similarly, Method of Levels therapy (MOL) (Carey, 2006) aims to increase clients’ sense of control by helping them to explore and increase their awareness of both sides of an internal conflict. This may increase suicidal clients’ awareness of goals underlying their reasons for living if the conflict which they explore is their ambivalence about suicide.

Our theoretical framework is also consistent with theoretical literature on schemas (Fredrickson, 2004; Johnson et al., 2008; Tarrier et al., 2013) which suggest that the activation of schemas related to “suicide as a means of escape” inhibits schemas containing more positive memories, thoughts, or emotions. Our framework conceptualizes this inhibition as an exclusive focus on the goal of ending one’s life, and narrowed awareness of memories, thoughts, and emotions related to one’s other goals. The BMAC task (Tarrier, 2010; Johnson et al., 2013; Tarrier et al., 2013), which was developed from cognitive models of suicide, is also consistent with our approach. The aim of the BMAC is to strengthen and build content of and access to positive schemas, so that clients become more aware of these experiences, appraisals, and coping strategies, and then are more able to access such schemas when experiencing a suicidal crisis (Johnson et al., 2013; Tarrier et al., 2013). The BMAC achieves this by encouraging clients to hold positive memories in their minds and explore and re-experience these positive memories and associated emotions (Johnson et al., 2013; Tarrier et al., 2013). The rationale for the BMAC is that if clients become able to access some positive material when in crisis, this initial activation will then enable the client to subsequently be able to access further positive material, since the triggering and content of the positive schema has been strengthened (Johnson et al., 2013). Our theoretical framework would interpret this as increasing clients’ awareness of goals other than death from suicide, and posits that once clients have a greater awareness of these other goals, they become less likely to avoid thinking about them.

### Differences in Ability to Imagine Consequences and Make Decisions

Suicide ideators and attempters differ in their ability to think through consequences of their actions and make decisions (Klonsky and May, 2010; Saffer and Klonsky, 2018), both of which involve mentally simulating future events (Gilbert and Wilson, 2007). Suicide attempters have a less specific memory retrieval style than non-attempters, and also demonstrate reduced specificity compared to non-attempters when imagining future events (Williams et al., 1996). This reduced ability to generate specific details when imagining future events may result in less specific content of “flash-forward” suicide imagery, since suicide imagery occurs by the same process by which individuals imagine future events (Williams et al., 1996; Holmes et al., 2007b). This in turn may influence whether individuals attempt suicide (Holmes et al., 2007b; Hales et al., 2011). Similarly, it may account for difficulties in solving problems which have been observed in suicidal individuals (Schotte and Clum, 1987). However, few of these findings have been replicated, which is partly due to the small number of studies comparing ideators with attempters, and inconsistency in their methods resulting from the use of different measures to assess the same constructs (Saffer and Klonsky, 2018). Moreover, existing theoretical models do not provide an explanation of the relationship between these psychological processes in terms of a common pathway, or outline a mechanism by which this common pathway could lead to suicide attempts. Refinements to the theoretical literature could provide a more in-depth explanation integrating these findings and specifying a common mechanism by which these psychological processes lead to suicide attempts. This would enable the development of a psychological tool to assess this mechanism, which could lead to greater consistency in future research on differences between ideators and attempters.

#### Psychological Differences Between Ideators and Attempters Reflect Limited Awareness

Our theoretical framework has potential to explain psychological differences which have been observed between ideators and attempters in existing literature. Firstly, reduced attentional control, including the ability to inhibit responses (Richard-Devantoy et al., 2015; Thompson and Ong, 2018), would be interpreted from a PCT perspective as a reduced ability to sustain or shift awareness between goals (Mansell et al., 2012). Consequently, individuals with reduced attentional control are likely to have more limited awareness of how suicide might negatively affect their other goals. In addition, differences in how ideators and attempters imagine events or consequences (Williams et al., 1996; Klonsky and May, 2010) are consistent with our hypotheses, since the ability to simulate future events would affect individuals’ awareness of the impact of suicide on their goals. These findings may also be linked to differences in ability to make decisions and solve problems (Schotte and Clum, 1987; Saffer and Klonsky, 2018), since both of these processes are likely to involve imagining future events (Gilbert and Wilson, 2007; Schacter et al., 2008, 2012; Schacter, 2012), and therefore may also affect awareness of consequences. In some cases, fearlessness about death observed in attempters (Smith et al., 2016) may also be due to limited awareness, since if individuals have limited awareness of goals which would be negatively affected by suicide, this may reduce their fear of death. Furthermore, there is emerging evidence from ongoing work to support the possibility of the new framework having validity (Wynford-Thomas et al., in preparation). When interviewed, individuals who had attempted suicide described having no awareness of potential consequences at the time of the attempt. In contrast, individuals who had only contemplated suicide were deterred by awareness of other goals, such as a desire to avoid upsetting family members.

#### Fluctuations in Awareness of Goals

Because awareness of goals fluctuates (Carey, 2006), individuals may experience more limited awareness of their goals on some days, yet have greater awareness on others. Therefore, our framework does not consider the process by which individuals attempt suicide to be a linear transition. Conversely, it predicts that individuals who experience suicidal ideation are most likely to attempt suicide at times when their awareness of how death from suicide would affect their higher-level goals is limited.

In some cases, individuals can swiftly move into a state of heightened shame and regret soon after a non-fatal suicide attempt (Wiklander et al., 2003), due to an increased awareness of the potential impact on their goals following the attempt. This may occur if the realization that they almost died leads the individual to imagine the consequences of their suicide that would have negatively impacted upon their personal goals. Conversely, individuals who go on to attempt suicide on multiple occasions would still have limited awareness following the initial attempt and consequently would not have imagined how suicide would affect their other goals.

Limited awareness depends on an individual’s mental flexibility; in other words, their ability and willingness to shift their awareness to and sustain their attention on other goals (Mansell et al., 2012). Mental flexibility can be affected by variables that impact on cognitive functioning, such as substance abuse or certain physiological states (Pompili et al., 2010). Following a suicide attempt, when the individual’s cognitive abilities are no longer affected by such variables, they may become more aware of consequences of suicide which would negatively impact upon their achievement of other goals. In addition, awareness of these negative consequences of suicide could be affected by the extent to which an individual believes that suicide will enable them to achieve the goal they have become focused on, and the importance of that goal. For example, Lucy became so focused on ending her life because she no longer felt able to tolerate the psychological distress she was experiencing, and believed that suicide would end this distress. This focus on suicide as a means of ending one’s distress is likely to be strengthened the more an individual tries but fails to find an alternative solution. However, the core prediction of our model is that regardless of the reason for the individual’s limited awareness, which vary with individual circumstances, it is always limited awareness that causes individuals to make a suicide attempt.

## Conclusions of the Review

We have described several ways in which existing theoretical ideas could be refined to integrate explanations of psychological processes that occur when individuals become suicidal and attempt suicide. These psychological processes can be grouped together into the broad headings of psychological processes which predispose individuals to suicidal crises, psychological processes which precipitate suicidal crises, and psychological processes which mediate suicide attempts. We have also introduced a new theoretical framework, and outlined the ways in which these existing theoretical concepts are integrated by the new framework. Lastly, we explained how, according to our theoretical framework, the concepts of control and goal conflict mediate a common pathway to suicide that underlies the psychological processes and risk factors which are included in previous theoretical models. This common pathway may provide a clearer treatment target, thereby allowing for the use of a more flexible, client-centered psychological intervention which can be used during immediate suicide crises. These claims will be discussed in the “Clinical Implications” section. The following section will propose novel hypotheses for future research.

## Hypotheses

According to our framework, we hypothesize that: (1) Individuals who contemplate and/or attempt suicide significantly differ from individuals who are not suicidal in terms of the level of goal conflict they report; (2) High levels of reported goal conflict predict psychological distress in individuals who contemplate and/or attempt suicide; (3) A reduction of goal conflict in suicidal individuals predicts a reduction in psychological states which are important components in recent theoretical models of suicide (e.g., perceived burdensomeness, thwarted belongingness, defeat, and entrapment); (4) Individuals who attempt suicide have a significantly reduced awareness of consequences of suicide which negatively impact on their important life goals, values, principles, or ideals, compared to individuals who contemplate suicide; (5) An intervention that increases suicidal individuals’ awareness of the impact of suicide on their goals will decrease the occurrences of suicide behaviors.

Hypotheses 1 and 2 could be tested using methodologies which have tested similar hypotheses on goal conflict in other clinical populations (Kelly et al., 2011a,b, 2013). A study which recruited individuals with depression (Kelly et al., 2011a) used the Strivings Instrumentality Matrix (SIM, 10 × 10 version) (Emmons and King, 1988) to assess conflict between their goals over time. The same measure and method could be used in suicidal individuals. Alternatively, Lauterbach’s Computerised Intrapersonal Conflict Assessment (CICA) (Lauterbach and Newman, 1999) could be used to assess conflict between individuals’ goals (Kelly et al., 2015). Further details on these methodologies for assessing goal conflict have been described elsewhere (Kelly et al., 2015). To test hypothesis 3, goal conflict could be assessed in a longitudinal study using one of these measures in addition to standardized measures of other key components of theoretical models of suicide, such as the Interpersonal Needs Questionnaire (INQ) (Orden et al., 2012), which assesses perceived burdensomeness and thwarted belongingness. The association between scores on these measures and goal conflict at various time points could be analyzed.

In order to test hypotheses 4 and 5, it would be necessary to develop and validate a tool for assessing individuals’ awareness of the impact of suicide on their goals. Ideators’ awareness of these consequences, as assessed by this tool, would then need to be compared with attempters’ awareness, in order to ascertain whether the two groups significantly differ. Lastly, it would be necessary to test the effect of an intervention that aims to increase suicide attempters’ awareness of the impact of suicide on their goals. A new program of research is currently ongoing, which aims to develop the tool for assessing awareness and test these hypotheses. During this program, data from cognitive interviews will be used to develop a clinical interview for assessing awareness (i.e., the awareness assessment tool), which can be scored quantitatively. This clinical interview will then be tested for reliability and validity among individuals who have contemplated or attempted suicide, and the two groups will be compared using the clinical interview once it has been developed. Once this research has been conducted, the clinical interview can be used to assess suicidal individuals’ awareness before and after an intervention that aims to increase awareness of their goals, such as Method of Levels therapy (Carey, 2006).

## Limitations of a PCT-Informed Theoretical Framework for Understanding Suicide

While the new theoretical framework has distinct strengths, it also has limitations. Firstly, since its theoretical constructs of control, conflict, and awareness are very broad, further work is needed to develop, refine, and adapt methodologies of testing hypotheses that are driven by these theoretical constructs. Previous research involving other clinical populations has made progress in developing and applying these methodologies (Kelly et al., 2011a,b, 2013, 2015), thereby contributing to an emerging evidence-base for the principles of PCT and their use in clinical populations. Furthermore, some methods will be adapted and tested in a suicidal population in an ongoing program of research. However, further work will be needed to provide further support for the application of these PCT principles and their associated methodologies in a suicidal population. Some of this work, in particular the development of accurate and useful assessments of these theoretical constructs in suicidal individuals, may be challenging since these theoretical constructs are so broad. For example, accurately assessing conflicting goals outside of individuals’ awareness may prove to be an empirical challenge. Secondly, since our theoretical approach is so novel, a substantial amount of research will be needed to establish its utility in a suicidal population. As part of this research, it will be necessary to rigorously test the effectiveness of interventions which are informed by this theoretical framework in larger scale studies with suicidal participants. Lastly, in cases when researchers are particularly interested in the relationship between specific risk factors and suicide attempts, the use of other theoretical approaches may be more appropriate, since our theoretical framework does not focus on the impact of specific risk factors.

## Clinical Implications

Our framework has direct clinical implications for treatment and risk assessment, and recommends the use of a therapy which has some distinct features. Firstly, our framework recommends the use of psychological interventions which specifically aim to reduce goal conflict and increase individuals’ awareness of their goals. This is distinct from interventions which target psychological states that are posited to arise due to goal conflict in our theoretical framework, such as perceived burdensomeness or hopelessness. We posit that the treatment targets of goal conflict and suicidal individuals’ awareness of their goals address their problems more directly than treating specific psychological states such as perceived burdensomeness. We also propose that simple and effective interventions which directly address these treatment targets may be particularly useful when suicidal clients are in immediate crisis. Furthermore, we posit that the distressing feelings (e.g., perceived burdensomeness) that are targeted by other interventions would be reduced as a result of resolution of the goal conflict. Since our framework emphasizes the importance of control of one’s experiences, this is also most consistent with therapeutic approaches which facilitate exploration of clients’ higher-level goals at their own pace. One such therapy which is specifically appropriate from the basis of this model, and already uses the questioning style which we suggest, is Method of Levels therapy (MOL) (Carey, 2006).

Method of levels is a transdiagnostic therapy which has been used with good effect with a variety of clinical populations, including individuals who have contemplated or attempted suicide (Bird et al., 2013; Carey et al., 2013; Griffiths et al., 2019a,b; Grzegrzolka et al., 2019). MOL is unique due to its focus on helping clients shift and sustain awareness to the source of goal conflicts, and its emphasis on facilitating clients’ control over the therapy session and their experiences (Carey, 2008). The approach used in MOL aims to directly address the problems of loss of control of one’s experiences and limited awareness which are described in this model, by increasing clients’ control and awareness of their goals. There is a variety of literature available elsewhere on how MOL achieves these aims, including concrete examples of its questioning style and the format of a therapy session (Carey, 2006; Mansell et al., 2012; Carey et al., 2015; Tai, 2016).

Several treatments currently exist which are specifically intended to reduce suicide risk and suicidality (Jobes et al., 2015), such as Dialectical Behavior Therapy (DBT) (Linehan et al., 1991), Cognitive Behavioral Therapy for Suicide Prevention (CBSP) (Tarrier et al., 2013), and the Collaborative Assessment and Management of Suicidality (CAMS) (Jobes, 2006). Brief interventions also exist, such as Motivational Interviewing for Suicidal Ideation (MI-SI) (Britton et al., 2012), the Teachable Moment Brief Intervention (TMBI) (O’Connor et al., 2015), and the Safety Planning Intervention (SPI) (Stanley and Brown, 2012). When compared with DBT, CBSP, and CAMS, MOL is a briefer, less structured intervention (Griffiths et al., 2019a,b), and does not involve multiple structured clinical assessments or skills training (Carey, 2006). This has the distinct advantages that it is efficient and can be adapted for use in challenging settings where there is limited time and resources, such as mental health wards, prisons, and A&E departments (Tai, 2009, 2016). Therefore, it is more similar to MI-SI, another brief less structured intervention which only requires one or two sessions (Britton et al., 2012). However, MOL has the advantage over MI-SI of having a more in-depth transdiagnostic theoretical basis which has been tested in other clinical populations (Kelly et al., 2011a; Mansell et al., 2014; Healey et al., 2017). Kovacs and Beck’s internal struggle hypothesis (Kovacs and Beck, 1977), which is the theoretical basis for MI-SI, posits that individuals attempt suicide when their wish to die is greater than their wish to live. PCT, the theoretical basis for MOL, expands on this account by conceptualizing an individual’s wish to die and wish to live as being affected by their higher-level goals and awareness of those goals (Powers, 1973).

Since MOL aims to be more flexible in its delivery than interventions such as DBT, CBSP, CAMS, the TMBI, and the SPI, this may facilitate suicidal individuals having more opportunities to speak freely about their problems at their own pace. This is an aspect of MOL which clients experiencing psychosis have reported to be helpful (Griffiths et al., 2019b). In addition, suicidal patients have expressed a need for more opportunities to speak freely about their problems, and benefitted from therapy which enables them to do so (Britton et al., 2012; Hagen et al., 2018). MOL also has similar advantages to CAMS of being individualized to that specific client and non-judgmental about their problems, since its curious questioning style facilitates an exploration of clients’ idiosyncratic problems, and discovery of solutions which are most suited to them (Mansell et al., 2012; Jobes, 2017). This meets the need expressed by suicidal patients on inpatient wards for more time spent by staff on exploring their individual problems in a non-judgmental way (Hagen et al., 2018). Lastly, individuals who make multiple suicide attempts have described surviving as an ambiguous state of indecision between wanting to die and wanting to live, and have benefitted from an increased sense of personal control (Bergmans et al., 2017). MOL has the potential to directly address this by enabling them to resolve this internal conflict by becoming more aware of goals related to their reasons for living, and increase their sense of control (Carey et al., 2015).

The use of our theoretical framework would also change how clinicians interpret the psychological processes underlying clients’ suicide risk. Clinicians already ask clients about their reasons for living (Berman and Silverman, 2014), but our framework would interpret this as assessing their awareness of how their other goals would be affected by suicide. For example, if a client’s reason for living is that they would no longer be able to look after their children if they died, this would be interpreted as the higher-level goal of “looking after my children.”

In contrast to therapeutic approaches that involve developing more reasons for living (e.g., Wenzel and Jager-Hyman, 2012), an approach to risk assessment and/or treatment based on our framework would involve enabling clients to become more fully aware of, and to more fully access the imagery, feelings and memories involved in, their existing higher-level goals. For example, the therapist could ask the client questions to explore thoughts and imagery they experience about suicide. If the client described imagery related to consequences which would negatively impact on their goals, the therapist would ask questions that encourage the client to focus on these consequences and explore what they mean from that client’s perspective. For example, if the client mentioned experiencing images of their family looking upset while they are feeling suicidal, the therapist might ask “What is it that bothers you about that?” or “What do you make of that?”. They might also ask the client about specific details of that mental image, such as which family members are present, what else is happening in the image, and whether that image is at the forefront or back of their mind. This has the aim of encouraging the client to focus on and become more aware of the higher-level goals which are related to that image, thus becoming more aware of consequences of suicide which would negatively impact upon that client.

Lastly, clinicians may wish to use structured risk assessments, such as ratings of reasons to die and reasons for living in CAMS (Jobes, 2006), or interventions such as the SPI, in addition to MOL. We do not believe this is necessary for therapeutic change to occur, since MOL aims to target a core process of therapeutic change (Griffiths et al., 2019a), thereby reducing distress and decreasing suicidality. However, some clinicians may prefer to do this or it may be necessary to meet the requirements of their service. In these cases, these assessments and/or interventions would not occur during an MOL session, and instead would either take place on a separate occasion or immediately prior to or after the MOL session.

## Conclusion

A more refined approach is needed to fully understand suicide, in particular the mechanism underlying suicide attempts. We propose that the common pathway underlying suicide attempts is an acute loss of sense of control of one’s experiences, combined with limited awareness of one’s personal goals, both of which are specific treatment targets. The application of this theoretical framework to the treatment of suicidal individuals involves the use of a brief, flexible, client-centered therapy with distinct advantages, which aims to directly address these treatment targets. Our theoretical framework offers potential refinements to existing theoretical literature and suggestions for integration of existing findings.

## Author Contributions

VM led the conceptualization of the theoretical ideas expressed in this article under the supervision of ST, WM, and DP. VM drafted the original manuscript with input from ST, WM, and DP. All authors contributed to the revision of the manuscript. All authors read and approved this final version of the manuscript.

## Conflict of Interest

The authors declare that the research was conducted in the absence of any commercial or financial relationships that could be construed as a potential conflict of interest.
